# Trial and Participant Characteristics of a Home-Visiting Diabetes Intervention: The *Together Overcoming Diabetes* Study

**DOI:** 10.1155/jdr/6591307

**Published:** 2025-04-24

**Authors:** Melissa L. Walls, Kelley J. Sittner, Gabby J. Gomez, Reagan E. Cole, Sylvie R. Perkins, Rachel I. Steinberg, Angie K. Forsberg, Emily E. Haroz, Allison Barlow

**Affiliations:** ^1^Department of International Health, Johns Hopkins University Bloomberg School of Public Health, Baltimore, Maryland, USA; ^2^Department of Sociology, Oklahoma State University, Stillwater, Oklahoma, USA

## Abstract

**Background:** American Indians (AIs) endure the most severe health inequities in the nation, including disproportionately high rates of Type 2 diabetes (T2D). We describe baseline characteristics for AI participants enrolled in a culturally grounded, intergenerational, home-based T2D preventive intervention called *Together Overcoming Diabetes* (TOD).

**Methods:** This community-based participatory research collaboration between five tribal nations and university-based researchers launched recruitment for a waitlist randomized control trial (RCT) design in 2021. Eligible participants were adults diagnosed with T2D who self-identified as AI, lived on or near participating reservations, and were caregivers to youth aged 10–16 years. Participants completed baseline assessments upon enrollment before being randomized to the intervention or waitlist group.

**Results:** A total of *N* = 162 individuals (81 adults and 81 youth) enrolled in the study. Most of the adult (Indigenous) sample reported being female (77.8%) and were on average 49.5 years old. Average age of youth participants was 13.2 years, with similar representation of girls and boys. Mean adult HbA1c (primary outcome for the trial) was 7.93 (SD = 1.99) at baseline. Around 19% of youth participants reported a T2D or prediabetes diagnosis. Additional demographic and holistic health results are presented.

**Conclusion:** This study provides comprehensive information about physiological, psychological, behavioral, and sociodemographic characteristics for a sample of AI families enrolled in a T2D intervention study. Findings suggest that intervention goals to improve behaviors like diet and physical activity are warranted and highlight the need for policy changes to address the social determinants of health.

**Trial Registration:** ClinicalTrials.gov identifier: NCT04734015

## 1. Introduction

American Indian and Alaska Native (AI/AN, also Indigenous) communities withstand extreme health inequities due to colonization and ongoing marginalization [1]. Premature mortality is highest among AI/AN Peoples in the United States relative to other groups: Indigenous persons born in 2022, for example, are projected to live 11.2 fewer years than White Americans born that same year [2]. Of relevance to this study, Type 2 diabetes (T2D) remains among the top five leading causes of death for AI/ANs [3]. While recent analyses suggest a possible plateau or decline in T2D prevalence among AI/AN adults seeking care in Indian Health Service (IHS) systems [4], AI/ANs remain nearly three times more likely to be diagnosed and 2.3 times more likely to die from T2D than are non-Hispanic Whites [5]. Youth onset T2D prevalence rates have risen over the past several decades [6] and are projected to continue growing through 2060 [7]. Initial evidence suggests heightened incidence of T2D among 10–19-year-old Americans following the COVID-19 pandemic [8]. Such forecasts are especially worrisome in that earlier onset T2D is associated with increased risk for severe health complications in later childhood and over the life course [9], and minoritized youth including AI/AN children bear a disproportionate and widening burden of T2D and its consequences [7, 10, 11].

It is thus important to generate useful data by and for AI/AN Peoples living with diabetes and support the development and evaluation of multigenerational, culturally relevant approaches to diabetes preventive intervention in collaboration with AI/AN communities. Existing adult diabetes prevention programs (DPPs) frequently target physical activity and diet and have been shown to be cost-effective and impactful for preventing T2D onset compared to pharmacological treatments [12]. The Special Diabetes Program for Indians (a prominent lifestyle intervention) long-term evaluation results indicate sustained reductions in weight and diabetes risk 10 years after baseline [13]. These are critical achievements; yet there remains room for health programming that addresses root causes of health inequities, promotes family well-being, and addresses barriers (e.g., transportation availability issues, cultural safety issues, and childcare needs) to care that remain heightened for many Indigenous Peoples.

For the current study, we worked as a university/community team to adapt and enhance a family-based home visiting program built by and for Indigenous communities (more information on this process and details about the intervention are available elsewhere) (M. [14]). Briefly, the intervention component of the *Together Overcoming Diabetes* (TOD) study was designed to address the root causes of T2D and its complications with Indigenous communities by promoting healthy lifestyles, activating culturally relevant coping resources and responses, increasing historical awareness, breaking generational cycles, and increasing knowledge that leads to improved holistic health. The intervention utilizes a home visiting model that removes transportation and childcare barriers to care, and the curriculum was designed and adapted for community and cultural contexts and multigenerational changes. As one example of enhancement, we aimed to address stress-coping factors and historical contexts of health inequities in the intervention curriculum. This is important given disruptions to Indigenous cultural ways, sustenance systems, and family dynamics due to colonization that have created heightened exposure to historical and intergenerational stress and trauma [15, 16].

The purpose of this study is to describe trial design and baseline characteristics for participants enrolled in TOD, a culturally grounded, intergenerational, home-based T2D preventive intervention. The baseline data presented here contribute to addressing a dearth of multigenerational data among AI families and contribute new information about AI physical, mental, and behavioral health.

## 2. Materials and Methods

The TOD study is a community-based participatory research (CBPR) collaboration between five Ojibwe communities and researchers at the Johns Hopkins Center for Indigenous Health. The team has worked in partnership for over a decade [17] on studies to understand how stress, mental health, and culturally salient coping strategies relate to T2D management in Ojibwe contexts. Our CBPR structure for collaborative governance involves Community Research Councils (CRCs) composed of community members including elders, service providers, and individuals living with T2D in each of the five tribal locations. Members of CRCs are actively involved in study planning, survey development, personnel decisions, and results dissemination. Our team delivers results to communities via written and verbal technical reports, online videos, and creative infographic summaries. All study procedures were reviewed and approved by the Johns Hopkins University IRB (IRB No. 10045), a Tribal Research Review Board, and the IHS National Institutional Review Board (IHS NIRB N20-N-01).

### 2.1. Trial Design and Participants

The TOD study includes a waitlist randomized control trial (RCT) design implemented on five reservations in Minnesota and Wisconsin. Eligible target participants were adults diagnosed with T2D who self-identified as AI, lived on or near (within 50 miles) participating reservations, and were caregivers to youth aged 10–16 years who were living in their home at the time of study screening. Because T2D control can become more difficult for patients with advanced disease, special exclusion criteria for adults (following protocols from DPP trials [18]) included (1) pregnancy, (2) end-stage renal disease on dialysis, and/or (3) any condition that would affect successful participation. We chose to focus enrollment on family dyads (adult/youth) to leverage cultural norms that promote caring for future generations with hopes that adult/child interactions could motivate family-based changes within the home and interrupt intergenerational health problems.

Recruitment for the trial occurred between 2021 and 2023. Strategies for recruitment included canvassing communities and tribal clinical facilities with study flyers, brochures, radio interviews, printed advertisements, and electronic interest form submission options. Prior to enrollment, study staff conducted eligibility screening of inclusion/exclusion criteria, including confirmation of a laboratory diagnosis of T2D via collaboration with local clinical facilities. Trained local (i.e., members of participating tribal communities) independent evaluators (IEs) led informed consent procedures prior to baseline assessment. Upon receipt of baseline data at university offices, enrolled contacts were randomized to immediate intervention or waitlist and notified of randomization status. Intervention families were then contacted by a trained local family health coach for intervention delivery.

**Figure 1:**
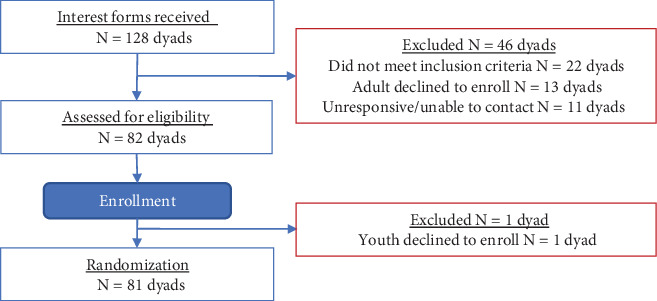
CONSORT diagram for TOD baseline enrollment.

A total of *N* = 162 individuals or *N* = 81 dyads (81 adults and 81 youth) enrolled in the study (Figure 1). Enrollment closed as of March 29, 2023.

### 2.2. Measures

We present select baseline measures in the current study. The primary outcome for the trial is HbA_1c_ and was measured using the CLIA-waived Afinion 2 analyzer, manufactured by Abbott. Five other physiological variables were assessed. Triglycerides and cholesterol were measured using the CLIA-waived Cholestech LDX analyzer, manufactured by Abbott. IEs used the Omron Digital Platinum Blood Pressure Monitor to measure blood pressure. IEs also collected measurements for weight (in pounds) and height (in feet and inches). We calculated BMI and BMI categories in Stata/SE 17 using the BMI program [19]. Participants were also asked to report the time since their T2D diagnosis,

We included three variables assessing psychological wellbeing. Depressive symptoms were assessed with the nine-item Patient Health Questionnaire [20]. Anxiety symptoms were assessed with the seven-item generalized anxiety disorder [21]. For both PHQ-9 and GAD-7, participants indicated the presence of symptoms in the past 2 weeks using a 4-point scale (0 = *not at all*, 1 = *several days*, 2 = *more than half the days*, 3 = *nearly every day*); mean scales were constructed by averaging the nine and seven items, respectively. In addition to the continuous measures of depressive and anxiety symptoms, we examined them at different symptom severity levels. For depressive symptoms, cut points of 5, 10, 15, and 20 correspond to mild, moderate, moderately severe, and severe symptoms [20]. For anxiety symptoms, cut points of 5, 10, and 15 correspond to mild, moderate, and severe [21]. We assessed wellbeing with an adapted version of the flourishing scale [22], with eight questions about self-perceived success in relationships, self-esteem, purpose, and optimism on a 5-point scale (1 = *strongly disagree*, 2 = *disagree*, 3 = *neither disagree nor agree*, 4 = *agree*, 5 = *strongly agree*). Responses were averaged to create an overall indicator of subjective wellbeing.

Behavioral variables assessed were physical activity, healthy eating behaviors, and diabetes medication use. Physical activity in the past week was measured with a question adapted from the National Health and Nutrition Examination Survey [23]. Participants were asked how many days in a typical week they engaged in any sports, fitness, or recreational activities. Participants who said they did not engage in any activities were coded as 0 days. Healthy eating behaviors were assessed with three separate measures. The first, sugar sweetened beverage (SSB) consumption, was measured using two items adapted from the Behavior Risk Factor Surveillance Survey [24]. Participants were asked what size of SSB was typically consumed (in ounces) in the past week and how many SSBs of that size were consumed in the past week. The second diet measure was assessed with two adapted questions [25]. Adult participants were asked how often they read nutrition labels on purchased foods and how often they prepared and cooked a main meal (0 = *never*, 1 = *rarely*, 2 = *sometimes*, 3 = *often*, 4 = *always*). The third diet measure concerned fast food intake. Adults were asked how many times in the past 7 days they had at least one meal or snack from a buffet or fast-food restaurant (0 = *0 times*; 1 = *1 to 3 times*; 2 = *4 or more times*). Medication use was a self-reported indicator of having a prescription for diabetes medication (0 = *no*, 1 = *yes*).

Youth physiological, psychological, and behavioral measures were collected using the same procedures as for adults, excluding measures requiring a blood draw (we did not collect blood for analysis from the youth). A standardized measure of BMI for youth that accounts for age and gender was calculated using the zanthro program in Stata/SE 17 [26]. Youth were also asked if they had ever received a prediabetes diagnosis or a T2D diagnosis (yes/no).

The current study includes the following adult demographic characteristics: income, age, gender, employment status, education, and relationship to youth in study. Adult personal income was measured by asking participants for their income in the past year from all sources. Response options were in $5000 ranges for respondents reporting income was less than $25,000 and in $10,000 ranges for income greater than $25,001. Responses were recoded to the midpoint of each range. Adult age was a continuous measure in years, calculated by subtracting their date of birth from the date of the interview. Adults indicated their gender as man, woman, or different identity. Adult participants indicated their employment status using the following response categories: employed full-time, employed part-time, student, retired, unemployed, disabled, and other. Education was measured by asking participants for their highest level of education. Adult participants were asked how they were related to the youth enrolled with them in the study. Demographic characteristics for youth enrolled in the study included age, education, and gender identity. Youth age in years was calculated by subtracting their date of birth from the date of the interview. Education was an indicator of the last grade they completed in school. Youth indicated whether they identify as girl, boy, or a different gender identity.

### 2.3. COVID-19 Impacts and Delays

Original plans were to launch the TOD trial in 2020. The COVID-19 pandemic delayed study commencement due to the home-visiting nature of assessments and intervention delivery and risk for disease spread. Project launch training occurred in the spring of 2021. Tribally mandated shutdowns ebbed and flowed across the communities through the first 2 years of the pandemic, and these factors slowed our ability to launch uniformly across all five communities. Throughout the trial, we worked closely with community teams, an external Data Safety and Monitoring Board, and program officials at the National Institutes of Health to assess participant safety, shift milestones, revise power calculations, and adjust sample size. The final revised goal for this trial was to enroll *N* = 75 dyads into the study at baseline.

### 2.4. Data Management and Quality Control

Survey data collected via the REDCap mobile app was uploaded by community-based IEs to the REDCap web platform hosted at the university within 24 h of completion. Data were screened for missing, inaccurate, or unclear responses by the study data manager within 24 h of upload and by a secondary screener within 3 days of upload. When the team identified errors, they followed up with IEs immediately for clarification and provided data quality reminders. Errors were corrected in REDCap if the IE provided clarification within 48 h of data collection.

### 2.5. Statistical Analysis

Descriptive statistics were used to examine the baseline sociodemographic characteristics and primary and secondary outcomes for the full sample of adults and youth enrolled in the study and for each group. The intervention and waitlist groups were compared using *χ*^2^ tests for categorical variables and *t*-tests for continuous variables.

## 3. Results

Table 1 displays sociodemographic characteristics of adult participants at baseline (e.g., those enrolled in the study between 2021 and 2023).

A majority of the adult (Indigenous) sample reported being female (77.8% overall), full-time employed (61.3%), and having at least a high school education. Overall, 18.8% of the adults reported being unemployed. Average age of the adult sample was 49.5 years (SD = 12). Most of the adults reported being a parent (57%) to the youth enrolled in the study with them.

Youth sociodemographic information is shown in Table 2. The average age of youth participants was 13.2 years, and most reported being in sixth grade at school. Similar numbers of boys and girls enrolled in the study, with two participants reporting a gender identity other than male or female. We found no significant differences in demographic characteristics between youth in the intervention versus waitlist group.

Select adult physiological, mental health, and behavioral variables are displayed in Table 3.

The mean baseline HbA_1c_ (primary trial outcome) value for the overall sample was 7.9, and average weight in pounds was 218.6. The mean reported duration since initial T2D diagnosis was 13.4 years (SD = 11.7). PHQ-9 cutoff scores indicate that 23% of the sample met criteria for mild depression and 21% reported moderate to severe depressive symptoms. Scores for the GAD-7 show that 27% of the baseline sample reported mild anxiety; 23.4% scored in the moderate to severe anxiety range. At the same time, adults on average reported high levels of wellbeing, with a sample mean of 4.1, (i.e., “agree” to flourishing statements). Adult participants reported being physically active an average of 1.5 days over the week prior to assessment, consumed on average 7.9 SSBs (i.e., a little more than one serving per day), and most (67.5%) reported eating fast food 1–3 times in the prior week. Results indicate that adults on average read nutrition labels sometimes (mean score = 1.9) and often prepare and cook meals (mean = 2.7). One measure in Table 3 was statistically distinct between the two study groups: Group Z reported significantly more days of physical activity in the past week than Group Y.

Select youth physiological, mental health, and behavioral variables are displayed in Table 4.

Nearly 13% of baseline youth said they had been diagnosed with prediabetes and close to 7% diagnosed with T2D. Mean BMI *Z*-scores overall were 1.85 (SD = 0.97), with nearly 20% of the youth categorized as overweight and 63.2% obese. Nearly one-third (31.3%) of the youth screened positive for mild depression and 35% met cutoff scores for moderate to severe depression. About 31% of the youth participants were classified with mild anxiety and 27.2% moderate to severe anxiety. Youth reported engaging in sports, fitness, or recreational activities approximately 3 days in the past week and consumed an average of about eight (7.9) SSBs in the week prior to their interview. Differences in physical activity reported between Groups Y and Z approached statistical significance, and we observed significant differences between groups on quantity of SSBs consumed. None of the remaining measures shown in Table 4 were significantly different at *p* < 0.05.

## 4. Discussion

This study provides comprehensive information about physiological, psychological, behavioral, and sociodemographic characteristics for a sample of AI adult/youth dyads enrolled in a T2D preventive intervention study using a randomized waitlist control design. Results demonstrate baseline equivalence between the treatment and waitlist groups for most variables collected in this study. We found significant differences between groups for reports of adult physical activity and youth SSB consumption that we will account for and explore further as follow-up assessment data spanning 24 months post baseline become available.

Adult participant eligibility requirements for the current study included verification of a T2D diagnosis. Comparing our findings to standards of care and guidelines for patients with diabetes [27, 28] demonstrates the need for preventive interventions in AI/AN communities *and* provides evidence of effective disease and comorbidity management in some cases. Mean HbA_1c_ values collected for this study were 7.9 (SD = 2), higher than both target goals for patients with T2D of < 7% [29] and estimated mean values (5.96) among AI adults in a multisite study of those eligible for DPP services [30]. Mean adult cholesterol values (LDL = 82.9 and HDL = 39.2) are on par with/very close to standard health recommendations (< 100 and > 40, respectively); however, recent ADA standards of care suggest LDL targets of < 70 for patients at higher risk for cardiovascular conditions [31]. Hypertension is a significant risk factor for additional comorbidities and disease complications among individuals living with T2D and is defined as systolic blood pressure ≥ 130 or a diastolic blood pressure ≥ 80 [29]. The mean blood pressure values obtained in this study correspond to hypertension, with significant variability in readings across participants (135/85, SD = 31.6 and 19.7). Relatedly, a study of AI and AN adults across 14 IHS units found that 77.9% of those living with diabetes also had hypertension [32].

Youth were enrolled in this trial alongside adults in our efforts to break cycles of chronic disease through intergenerational prevention strategies; thus, youth inclusion criteria did not require a diabetes or prediabetes diagnosis. Despite this, a notable proportion of youth (mean age = 13.2 years) reported having been told by a doctor that they had prediabetes (12.8%) or T2D (6.6%). Recent evidence suggests that 1 in 5 adolescents (around 18%) nationally have prediabetes [33]. These national results are based on laboratory analyses whereas we relied on youth self-reports, and many youths in our study may not have ever been clinically screened for diabetes or prediabetes (i.e., our results may be conservative estimates). A major risk factor for prediabetes and T2D is obesity; for instance, in a sample of AI children, those characterized as living with severe obesity showed a two- to fivefold increase in risk of developing T2D before age 20 compared to youth with lower levels of obesity [34]. Concerning, then, is that most of the AI youth in this study had zBMI scores equivalent to overweight (19.7%) or obese (63%) categories. Current diabetes care standards underscore the cost-effectiveness and positive impacts of DPPs [27], and small to moderate weight loss was associated with reduced diabetes risk in a 10-year follow-up of AI/ANs in a diabetes prevention study nationally [13]. Our waitlist control RCT design means that all families enrolled in the current trial will be invited to participate in TOD programming, with hopeful positive impacts on these youths' physiological outcomes.

A robust literature indicates that mental health plays a critical role in the onset and progression of T2D, and T2D can negatively impact mental health [35–37]. In this study, around 44% of adult participants living with T2D reported some degree (mild to severe) of depression and over half (50.6%) met cutoff scores for mild to severe anxiety. Youth mental health problems were higher than those reported by their adult family members: 66.3% of youth reported mild to severe depression and 58% mild to severe anxiety. These findings correspond to pressing inequities in mental health for some Indigenous youth and adults [38], though rates vary considerably across communities [39], and rising mental health issues among youth nationally, particularly in the wake of the global COVID-19 pandemic [40]. We simultaneously found that adults reported high levels of flourishing, an indicator of subjective wellbeing. This finding is on par with other reports demonstrating disproportionately high levels of Indigenous psychological wellbeing [41] relative to other groups [42] and is important for balancing deficit narratives and celebrating Indigenous strengths. At the same time, addressing mental health challenges is critical. As such, one aim of the TOD curriculum is to provide family-centered, culturally grounded resources for mental wellbeing and stress management tools to effectively address stressors known to impact mental health.

Limitations of this study include potential sample biases. It may be that families already motivated to address health were drawn to the current study; as such, characteristics of participants reported here could be conservative estimates of health conditions relative to communities overall. Another factor that may yield conservative results in this study is that some of our outcomes rely on self-reports of behaviors or health conditions (especially for youth). A strength of this study is that it provides regional estimates of T2D health profiles for AI community members. This is important given historic and ongoing calls for within-culture/locally relevant research attuned to the diversity of AI diabetes experiences across the nearly 600 federally recognized tribes in the United Sates. For example, data spanning 2006–2017 reveals considerable differences in the prevalence of diabetes across tribal regions. Indigenous Peoples in Alaska experienced the lowest rates (around 6%) and AIs in the Southwestern United States the highest (around 20% across years) [4]. Although demographic and health profiles vary across communities, *approaches* to diabetes prevention and management can be “scaled up” for cross-cultural sharing [43] and regional studies can inform equitable and impactful policy and practice decisions.

This study was designed in part because lifestyle interventions are an important tool for improved quality of life and disease outcomes for families living with T2D, yet there remains need for culturally relevant, multigenerational approaches. Our findings reveal room for improvement in the physiological and psychological domains discussed above for the Midwestern Tribal Nations engaged in this study, and we found evidence that intervention goals to improve behaviors like diet and physical activity are warranted. Importantly, the health outcomes and behaviors for Indigenous families revealed here emerge from deep social and structural determinants of health. Classic social determinants like income, education, and employment levels are lower in this study than national averages [44, 45] and are drivers of worse health status. Further, these proximal determinants of health emerge from longstanding oppressive policies and intergenerational impacts of historical trauma rooted in settler colonialism [1, 15]. In sum, uniquely profound issues of social injustice underpin T2D experiences in Indigenous communities. While the TOD curriculum explicitly incorporates problem-solving and coping techniques, culturally grounded teachings, and critical information about the historical context of Indigenous health, the root causes of health inequities are not easily addressed by behavioral interventions and signal need for policy change and education to redress the lingering impacts of colonial subjugation and support Indigenous Peoples in their efforts to reclaim culture and wellbeing.

## Figures and Tables

**Table 1:** Descriptive statistics, baseline sociodemographic characteristics—adults (*n* = 81).

	**Sample**	**Group Y**	**Group Z**	**p**
Income, $, mean ± SD (80 respondents)	$33,750 ± $21,736	$33,205 ± $20,431	$34,268 ± $23,152	0.829
Age, years, mean ± SD (81 respondents)	49.50 ± 11.97	48.71 ± 12.22	50.28 ± 11.83	0.559
Gender, % female (81 respondents)	77.78%	80.00%	75.61%	0.635
Employment, *n* (%) (80 respondents)				0.685
Full-time	49 (61.25%)	26 (66.67%)	23 (56.10%)	
Part-time	4 (5.00%)	1 (2.56%)	3 (7.32%)	
Student	1 (1.25%)	0 (0.00%)	1 (2.44%)	
Retired	5 (6.25%)	2 (5.13%)	3 (7.32%)	
Unemployed	15 (18.75%)	6 (15.38%)	9 (21.95%)	
Disabled	2 (2.5%)	1 (2.56%)	1 (2.44%)	
Other	4 (5.00%)	3 (7.69%)	1 (2.44%)	
Education, *n* (%) (80 respondents)				0.315
< HS	7 (8.75%)	1 (2.56%)	6 (14.63%)	
HS or GED	23 (28.75%)	10 (25.64%)	13 (31.71%)	
Some college	33 (41.25%)	19 (48.72%)	14 (34.15%)	
College	15 (18.75%)	8 (20.51%)	7 (17.07%)	
Advanced degree	2 (2.50%)	1 (2.56%)	1 (2.44%)	
Relationship to youth, *n* (%) (79 respondents)				0.618
Parent	45 (56.96%)	22 (55.00%)	23 (58.97%)	
Step-parent	1 (1.27%)	1 (2.50%)	0 (0.00%)	
Great-grandparent	2 (2.53%)	1 (2.50%)	1 (2.56%)	
Grandparent	22 (27.85%)	13 (32.50%)	9 (23.08%)	
Uncle/aunt	4 (5.06%)	2 (5.00%)	2 (5.13%)	
Legal guardian	5 (6.33%)	1 (2.50%)	4 (10.26%)	

**Table 2:** Descriptive statistics, baseline demographic characteristics—youth (*n* = 80).

	**Sample**	**Group Y**	**Group Z**	**p**
Age, years, mean ± SD (80 respondents)	13.24 ± 1.99	13.14 ± 2.13	13.34 ± 7.08	0.660
Grade in school, mean ± SD (80 respondents)	6.46 ± 2.01	6.33 ± 2.27	6.59 ± 1.75	0.579
Gender, *n* (%) (80 respondents)				0.250
Boy	38 (47.50%)	21 (53.85%)	17 (41.46%)	
Girl	50 (50.00%)	18 (46.15%)	22 (53.66%)	
Different identity	2 (2.50%)	0 (0.00%)	2 (4.88%)	

**Table 3:** Descriptive statistics, adult baseline outcomes (*n* = 81).

	**Sample mean**	**Group Y**	**Group Z**	**p**
Time since DX, years, mean ± SD (76 respondents)	13.41 ± 11.71	14.47 ± 10.31	12.41 ± 12.95	0.447
HbA1c, mean ± SD (80 respondents)^a^	7.93 ± 1.99	8.20 ± 2.07	7.67 ± 1.90	0.234
Triglycerides, mean ± SD (70 respondents)	201.10 ± 91.34	199.03 ± 92.82	203.06 ± 91.20	0.855
Total cholesterol, mean ± SD (70 respondents)	154.20 ± 40.10	151.65 ± 40.11	156.61 ± 40.50	0.608
HDL, mean ± SD (68 respondents)	39.15 ± 13.24	39.36 ± 13.98	38.94 ± 12.71	0.897
LDL, mean ± SD (58 respondents)	82.86 ± 31.64	85.86 ± 28.87	80.84 ± 34.05	0.594
Systolic blood pressure, mean ± SD (81 respondents)	135.41 ± 19.67	138.35 ± 17.26	132.54 ± 21.60	0.185
Diastolic blood pressure, mean ± SD (81 respondents)	84.62 ± 11.77	86.70 ± 11.89	82.59 ± 11.44	0.116
BMI, mean ± SD (80 respondents)	36.64 ± 9.08	37.47 ± 9.62	35.84 ± 8.58	0.427
BMI categories, *n* (%) (80 respondents)				0.265
Normal range	3 (3.75%)	1 (2.56%)	2 (4.88%)	
Overweight	14 (17.50%)	9 (23.08%)	5 (12.20%)	
Obese Class I	26 (32.50%)	9 (23.08%)	17 (41.46%)	
Obese Class II	12 (15.00%)	5 (12.82%)	7 (17.07%)	
Obese Class III	25 (31.25%)	15 (38.46%)	10 (24.39%)	
Weight, pounds, mean ± SD (80 respondents)	218.56 ± 55.59	223.70 ± 62.88	213.66 ± 47.93	0.423
Depressive symptoms, mean ± SD (81 respondents)	5.81 ± 5.39	6.10 ± 5.29	5.54 ± 5.54	0.641
Depressive symptom severity, *n* (%) (81 respondents)				0.901
Mild	19 (23.46%)	9 (22.50%)	10 (24.39%)	
Moderate	11 (13.58%)	7 (17.50%)	4 (9.76%)	
Moderately severe	4 (4.94%)	2 (5.00%)	2 (4.88%)	
Severe	2 (2.47%)	1 (2.50%)	1 (2.44%)	
Wellbeing, mean ± SD (80 respondents)	4.11 ± 0.57	4.05 ± 0.57	4.15 ± 0.58	0.420
Anxiety symptoms, mean ± SD (81 respondents)	6.21 ± 5.79	6.08 ± 5.89	6.34 ± 5.75	0.837
Anxiety symptom severity, *n* (%) (81 respondents)				0.819
Mild	22 (27.16%)	9 (22.50%)	13 (31.71%)	
Moderate	10 (12.35%)	5 (12.50%)	5 (12.20%)	
Severe	9 (11.11%)	5 (12.50%)	4 (9.76%)	
Days in past week being physically active, mean ± SD (79 respondents)	1.54 ± 2.27	0.87 ± 1.80	2.17 ± 2.50	0.010
Sugar sweetened beverages				
Typical size past week, ounces, mean ± SD (80 respondents)	11.38 ± 10.63	13.38 ± 12.97	9.46 ± 7.47	0.100
Quantity past week, mean ± SD (56 respondents)	7.89 ± 8.18	9.31 ± 9.45	6.37 ± 6.38	0.181
Read nutrition labels, mean ± SD (79 respondents)	1.89 ± 1.28	1.82 ± 1.19	1.95 ± 1.38	0.656
Prepare and cook meals, mean ± SD (80 respondents)	2.73 ± 1.11	2.56 ± 1.10	2.88 ± 1.12	0.210
Reported eating fast food past week, *n* (%) (80 respondents)				0.672
Never	13 (16.25%)	6 (15.38)	7 (17.07%)	
1 to 3 times	54 (67.50%)	28 (71.79%)	26 (53.41%)	
4 or more times	13 (16.25%)	5 (12.82%)	8 (19.51%)	
Medication use, *n* (% yes) (80 respondents)	73 (92.41%)	34 (89.47%)	39 (90.12%)	0.344

*Note:p* values for *F* and *χ*^2^ tests comparing Group Y and Group Z.

Abbreviation: DX, diagnosis.

^a^Primary outcome.

**Table 4:** Descriptive statistics, youth baseline outcomes (*n* = 80).

	**Sample mean**	**Group Y**	**Group Z**	**p**
Prediabetes diagnosis, *n* (%) (78 respondents)	10 (12.82%)	3 (7.69%)	7 (17.95%)	0.176
Diabetes diagnosis, *n* (%) (76 respondents)	5 (6.58%)	3 (7.89%)	2 (5.26%)	0.644
zBMI, mean ± SD (76 respondents)	1.85 ± 0.96	1.81 ± 1.02	1.90 ± 0.91	0.684
zBMI categories, *n* (%)				0.641
Underweight	1 (1.32%)	0 (0.00%)	1 (2.56%)	
Normal weight	12 (15.78%)	7 (18.92%)	5 (12.82%)	
Overweight	15 (19.74%)	8 (21.62%)	7 (17.95%)	
Obese	48 (63.16%)	22 (59.48%)	26 (66.67%)	
Systolic blood pressure, mean ± SD (81 respondents)	117.05 ± 17.95	117.98 ± 21.09	116.15 ± 14.46	0.650
Diastolic blood pressure, mean ± SD (81 respondents)	73.78 ± 12.85	74.58 ± 14.55	73.00 ± 11.08	0.585
Depressive symptoms, mean ± SD (80 respondents)	7.38 ± 5.40	8.31 ± 6.22	6.49 ± 4.37	0.132
Depression symptom severity, *n* (%) (80 respondents)				0.259
Mild	25 (31.25%)	9 (23.08%)	16 (39.02%)	
Moderate	19 (23.75%)	10 (25.64%)	9 (21.95%)	
Moderately severe	7 (8.75%)	5 (12.82%)	2 (4.88%)	
Severe	2 (2.50%)	2 (5.13%)	0 (0.00%)	
Anxiety symptoms, mean ± SD (81 respondents)	6.28 ± 5.00	6.30 ± 4.90	6.27 ± 5.15	0.977
Anxiety symptom severity, *n* (%) (81 respondents)				0.963
Mild	25 (30.86%)	13 (32.50%)	12 (29.27%)	
Moderate	17 (20.99%)	8 (20.00%)	9 (21.95%)	
Severe	5 (6.17%)	2 (5.00%)	3 (7.32%)	
Days in past week being physically active, mean ± SD (80 respondents)	3.08 ± 2.47	3.03 ± 2.44	3.12 ± 2.52	0.863
Sugar sweetened beverages				
Typical size past week, ounces, mean ± SD (80 respondents)	16.58 ± 9.97	18.15 ± 11.77	15.07 ± 7.75	0.169
Quantity past week, mean ± SD (53 respondents)	4.50 ± 1.87	5.04 ± 2.03	3.94 ± 1.54	0.032

*Note:* Youth who identified other gender not included in zBMI numbers. *p* values for *F*-test.

## Data Availability

Research data are not shared.

## References

[1] King M., Smith A., Gracey M. (2009). Indigenous Health Part 2: The Underlying Causes of the Health Gap. *Lancet*.

[2] Arias E., Tejada-Vera B., Kochanek K., Ahmad F. B. (2022). Provisional Life Expectancy Estimates for 2021. https://www.cdc.gov/nchs/data/vsrr/vsrr023.pdf.

[3] Heron M. (2021). Deaths: Leading Causes for 2018. https://www.cdc.gov/nchs/data/nvsr/nvsr70/nvsr70-04-508.pdf.

[4] Bullock A., Sheff K., Hora I. (2020). Prevalence of Diagnosed Diabetes in American Indian and Alaska Native Adults, 2006–2017. *BMJ Open Diabetes Research & Care*.

[5] US Department of Health and Human Services (2018). Diabetes and American Indians/Alaska Natives|Office of Minority Health. https://minorityhealth.hhs.gov/diabetes-and-american-indiansalaska-natives.

[6] Perng W., Conway R., Mayer-Davis E., Dabelea D. (2023). Youth-Onset Type 2 Diabetes: The Epidemiology of an Awakening Epidemic. *Diabetes Care*.

[7] Tönnies T., Brinks R., Isom S. (2022). Projections of Type 1 and Type 2 Diabetes Burden in the U.S. Population Aged <20 Years Through 2060: The SEARCH for Diabetes in Youth Study. *Diabetes Care*.

[8] Mefford M. T., Wei R., Lustigova E., Martin J. P., Reynolds K. (2023). Incidence of Diabetes Among Youth Before and During the COVID-19 Pandemic. *JAMA Network Open*.

[9] Dabelea D., Stafford J. M., Mayer-Davis E. J. (2017). Association of Type 1 Diabetes vs Type 2 Diabetes Diagnosed During Childhood and Adolescence With Complications During Teenage Years and Young Adulthood. *JAMA*.

[10] Mayer-Davis E. J., Lawrence J. M., Dabelea D. (2017). Incidence Trends of Type 1 and Type 2 Diabetes Among Youths, 2002–2012. *New England Journal of Medicine*.

[11] Writing Group for the SEARCH for Diabetes in Youth Study Group (2007). Incidence of Diabetes in Youth in the United States. *JAMA*.

[12] Diabetes Prevention Program Research Group (2009). 10-Year Follow-Up of Diabetes Incidence and Weight Loss in the Diabetes Prevention Program Outcomes Study. *Lancet*.

[13] Jiang L., Johnson A., Pratte K. (2018). Long-Term Outcomes of Lifestyle Intervention to Prevent Diabetes in American Indian and Alaska Native Communities: The Special Diabetes Program for Indians Diabetes Prevention Program. *Diabetes Care*.

[14] Walls M., Chambers R., Begay M. (2022). Centering the Strengths of American Indian Culture, Families and Communities to Overcome Type 2 Diabetes. *Frontiers in Public Health*.

[15] Walls M. (2023). The Perpetual Influence of Historical Trauma: A Broad Look at Indigenous Families and Communities in Areas Now Called the United States and Canada. *International Migration Review*.

[16] Walls M. L., Whitbeck L. B. (2012). Advantages of Stress Process Approaches for Measuring Historical Trauma. *American Journal of Drug and Alcohol Abuse*.

[17] Elm J. H. L., Handeland T. (2019). Momentum and longevity for tribally driven health equity science: evidence from the gathering for health project. *Human Biology*.

[18] Jiang L., Manson S. M., Beals J. (2013). Translating the Diabetes Prevention Program Into American Indian and Alaska Native Communities: Results From the Special Diabetes Program for Indians Diabetes Prevention Demonstration Project. *Diabetes Care*.

[19] Linden A. (2019). *BMI: Stata Module to Compute Body Mass Index*.

[20] Kroenke K., Spitzer R. L., Williams J. B. W. (2001). The PHQ-9. *Journal of General Internal Medicine*.

[21] Spitzer R. L., Kroenke K., Williams J. B. W., Löwe B. (2006). A brief measure for assessing generalized anxiety disorder: the GAD-7. *Archives of Internal Medicine*.

[22] Diener E., Wirtz D., Tov W. (2010). New Well-Being Measures: Short Scales to Assess Flourishing and Positive and Negative Feelings. *Social Indicators Research*.

[23] Centers for Disease Control and Prevention (CDC), National Center for Health Statistics (NCHS) (2017). *National Health and Nutrition Examination Survey Questionnaire*.

[24] Centers for Disease Control and Prevention (CDC) (2013). *Behavioral Risk Factor Surveillance System Survey Questionnaire*.

[25] Eisenberg D. M., Righter A. C., Matthews B., Zhang W., Willett W. C., Massa J. (2017). Feasibility Pilot Study of a Teaching Kitchen and Self-Care Curriculum in a Workplace Setting. *American Journal of Lifestyle Medicine*.

[26] Vidmar S., Carlin J., Hesketh K., Cole T. (2004). Standardizing Anthropometric Measures in Children and Adolescents with New Functions for Egen. *Stata Journal*.

[27] American Diabetes Association (2023). Introduction and Methodology: Standards of Care in Diabetes-2023. *Diabetes Care*.

[28] Lipman M. L., Schiffrin E. L. (2012). What Is the Ideal Blood Pressure Goal for Patients With Diabetes Mellitus and Nephropathy?. *Current Cardiology Reports*.

[29] ElSayed N. A., Aleppo G., Aroda V. R. (2023). 6. Glycemic Targets: Standards of Care in Diabetes—2023. *Diabetes Care*.

[30] Herman W. H., Ma Y., Uwaifo G. (2007). Differences in A1C by Race and Ethnicity Among Patients With Impaired Glucose Tolerance in the Diabetes Prevention Program. *Diabetes Care*.

[31] American Diabetes Association (2004). Dyslipidemia Management in Adults With Diabetes. *Diabetes Care*.

[32] Huyser K. R., Rockell J., Wilson C., Manson S. M., O’Connell J. (2020). Healthcare Utilization, Diabetes Prevalence, and Comorbidities: Examining Sex Differences Among American Indian and Alaska Native Peoples. *Race, Ethnicity, Gender and Other Social Characteristics as Factors in Health and Health Care Disparities*.

[33] Andes L. J., Cheng Y. J., Rolka D. B., Gregg E. W., Imperatore G. (2020). Prevalence of Prediabetes Among Adolescents and Young Adults in the United States, 2005-2016. *JAMA Pediatrics*.

[34] Tanamas S. K., Reddy S. P., Chambers M. A. (2018). Effect of Severe Obesity in Childhood and Adolescence on Risk of Type 2 Diabetes in Youth and Early Adulthood in an American Indian Population. *Pediatric Diabetes*.

[35] Aikens J. E. (2012). Prospective Associations Between Emotional Distress and Poor Outcomes in Type 2 Diabetes. *Diabetes Care*.

[36] Feng X., Astell-Burt T. (2017). Impact of a Type 2 Diabetes Diagnosis on Mental Health, Quality of Life, and Social Contacts: A Longitudinal Study. *BMJ Open Diabetes Research and Care*.

[37] Walls M. L., Sittner K. J., Aronson B. D., Forsberg A. K., Whitbeck L. B., Al’Absi M. (2017). Stress Exposure and Physical, Mental, and Behavioral Health Among American Indian Adults With Type 2 Diabetes. *International Journal of Environmental Research and Public Health*.

[38] Walls M., Sittner K. J., Whitbeck L. B. (2021). Prevalence of Mental Disorders From Adolescence Through Early Adulthood in American Indian and First Nations Communities. *International Journal of Mental Health and Addiction*.

[39] Novins D. K., Beals J., Roberts R. E., Manson S. M. (1999). Factors Associated With Suicide Ideation Among American Indian Adolescents: Does Culture Matter?. *Suicide and Life-threatening Behavior*.

[40] Samji H., Wu J., Ladak A. (2022). Review: Mental Health Impacts of the COVID-19 Pandemic on Children and Youth – A Systematic Review. *Child and Adolescent Mental Health*.

[41] Kading M. L., Hautala D. S., Palombi L. C., Aronson B. D., Smith R. C., Walls M. L. (2015). Flourishing American Indian Positive Mental Health. *Society and Mental Health*.

[42] Walls M., Pearson C., Kading M., Teyra C. (2016). Psychological Wellbeing in the Face of Adversity Among American Indians: Preliminary Evidence of a New Population Health Paradox?. *Annals of Public Health and Research*.

[43] Walters K., Walls M., Dillard D., Kaur J. (2019). *American Indian and Alaska Native Research in the Health Sciences: Critical Considerations for the Review of Research Applications*.

[44] US Bureau of Labor Statistics (2024). Labor Force Statistics From the Current Population Survey. https://www.bls.gov/cps/.

[45] US Census Bureau (2023). Income in the United States: 2022. https://www.census.gov/library/publications/2023/demo/p60-279.html.

